# Mechanical Performance Evaluation of Self-Compacting Concrete with Fine and Coarse Recycled Aggregates from the Precast Industry

**DOI:** 10.3390/ma10080904

**Published:** 2017-08-04

**Authors:** Sara A. Santos, Pedro R. da Silva, Jorge de Brito

**Affiliations:** 1 Civil Engineering Master, Instituto Superior Técnico, Universidade de Lisboa, 1049-001 Lisbon, Portugal; sara.a.santos@ist.utl.pt; 2 CERIS, Instituto Superior de Engenharia de Lisboa, Instituto Politécnico de Lisboa, 1959-007 Lisbon, Portugal; 3 CERIS, Instituto Superior Técnico, Universidade de Lisboa, 1049-001 Lisbon, Portugal; jb@civil.ist.utl.pt

**Keywords:** self-compacting concrete, coarse and fine recycled aggregates from the precast industry, fresh properties, mechanical properties

## Abstract

This paper intends to evaluate the feasibility of reintroducing recycled concrete aggregates in the precast industry. The mechanical properties of self-compacting concrete (SCC) with incorporation of recycled aggregates (RA) (coarse recycled aggregates (CRA) and fine recycled aggregates (FRA)) from crushed precast elements were evaluated. The goal was to evaluate the ability of producing SCC with a minimum pre-established performance in terms of mechanical strength, incorporating variable ratios of RA (FRA/CRA%: 0/0%, 25/25%, 50/50%, 0/100% and 100/0%) produced from precast source concretes with similar target performances. This replication in SCC was made for two strength classes (45 MPa and 65 MPa), with the intention of obtaining as final result concrete with recycled aggregates whose characteristics are compatible with those of a SCC with natural aggregates in terms of workability and mechanical strength. The results enabled conclusions to be established regarding the SCC’s produced with fine and coarse recycled aggregates from the precast industry, based on its mechanical properties. The properties studied are strongly affected by the type and content of recycled aggregates. The potential demonstrated, mainly in the hardened state, by the joint use of fine and coarse recycled aggregate is emphasized.

## 1. Introduction

The consumption of natural resources in the construction industry has been growing in the last decades, as well as the production of demolition, construction and rehabilitation works waste. This process of “construction-demolition” has exerted great pressure on natural resources, especially the natural aggregates (NA), and generated high levels of the so-called construction and demolition waste (CDW), often ending up in landfills. Both these aspects have a negative impact on the environment. Because of the present economic and environmental crisis, the demands for a more sustainable development in the construction sector require a different approach to the concrete technology.

The use of recycled aggregates (RA) to produce new concrete seems to be a potential solution. It could solve the problems related with storage, transportation and dumping of CDW and contribute to the sustainability of the environment by adding value to this waste and consequently limiting the consumption of NA.

On the other hand, since the capacity to satisfy the clients’ needs and the relationship performance-quality-cost are the main distinctive factors of a healthy competition in the construction sector, the study of self-compacting concrete (SCC) with RA must contribute to an approach between the technological development activities and the needs of both the construction industry and its final clients.

The presence of adhered mortar in RA is one of the main reasons for their loss of quality. Consequently, RA have lower density, greater water absorption and lower mechanical strength than the NA. These properties are the ones that most influence the performance of the SCC in which they are incorporated [1].

It is very important to evaluate the SCC fresh-state properties, since it must have specific characteristics to be considered self-compacting, i.e., it must have the capacity to flow and compact under its own weight only, fill the moulds with all the reinforcement bars, piping, negatives, etc., maintaining its homogeneity, and without the need of any on site vibration means [2].

Table 1 presents a summary of all studies analyzing the influence of the incorporation of RA (at an incorporation ratio of 100%) on the SCC fresh-state properties.

The fluidity of SCC was evaluated through the slumpflow diameter. In the studies analyzed, this parameter decreases for high coarse recycled aggregates (CRA) incorporation ratios, since they absorb more water than the coarse natural aggregates (CNA) [3,4]. Safiuddin et al. [5], Tuyan et al. [6] and Modani and Mohitkar [7] found that the slump-flow diameter increases for limited CRA incorporation ratios (less than 20% to 60%).

The viscosity of SCC is evaluated through the V-funnel flow time. It was found that it increases for high CRA incorporation ratios because of their rougher surface and more angular shape relative to CNA. Safiuddin et al. [5] and Modani and Mohitkar [7] found that the V-funnel flow time decreases for limited CRA incorporation ratios (less than 20% to 40%).

In terms of the SCC’s passing ability through confined spaces evaluated through the L-box passing ability index (PL), it was found that the PL values increase with the RA incorporation ratio. This was justified by the lower water absorption of the FRA in the beginning of the mixing [8], a higher W/C ratio [3] or an increase of the Superplasticizer (S_p_) content [6].

In terms of the SCC’s segregation resistance evaluated through the segregation ratio (SR), three different trends occur. Kou and Poon [8] found that the SR increased with the FRA incorporation ratio because of their higher water absorption capacity. On the other hand, Grdic et al. [3] found that the SR decreased as the CRA incorporation ratio grew due to the increase of water absorption. Finally, Safiuddin et al. [5] found that the SR increased for small replacement ratios of NA with RA (30% and 40%), but decreased for high ratios (70% and 100%), because the fines fraction increases when the CRA are fractured during the mixing process.

In terms of the SCC’s passing ability through confined spaces evaluated through the J-ring slump flow diameter, it was found that the slump-flow diameter increases for small CRA incorporation ratios (30% and 40%). For higher ratios (70% and 100%), the J-ring slump flow diameter decreases, because of the fracture into smaller particles of the CRA during the mixing process, leading to greater water absorption of the RA.

In the hardened state, it was found that the SCC’s bulk density at 28 days decreased around 3% for 100% CRA incorporation [4] and the ultrasonic pulse velocity (UPV) through SCC decreased around 6% for 60% CRA incorporation [6]. The reduction in these two properties (bulk density and UPV) was justified by the lower density of the RA relative to the NA.

Based on the works analysed, Table 2 presents a summary of the influence of the incorporation of RA (for 100% ratio) on the hardened-state SCC’s properties.

As seen in Table 2, the compressive strength and the flexural tensile strength decrease in most of the studies as the RA incorporation ratio increases because of their lower mechanical strength relative to the NA.

The incorporation of CRA reduces the modulus of elasticity [4]. The decrease is approximately 5% for 100% incorporation. This was justified by the lower stiffness of the CRA (relative to the CNA), given the presence of old mortar adhered to the original NA and the lower deformability of this mortar.

Shrinkage increases with the FRA incorporation ratio [8]. This was explained by the greater porosity of the FRA and consequent lower density (because of the hardened mortar contained in the FRA), which decrease their stiffness and capacity to restrict deformation.

Table 3 presents a summary of the results of the studies analysed on the influence of the incorporation of RA (for a ratio of 100%) on the durability properties of SCC.

Modani and Mohitkar [7] found that the SCC’s capillary water absorption increased with the W/C ratio and decreased as the CRA incorporation ratio grew, similarly to the water absorption by immersion [3]. This was justified by the greater water absorption of the CRA.

On the other hand, Pereira-de-Oliveira et al. [4] found that the capillary water absorption decreases as the replacement of CNA with CRA increased. This was justified by the high volume of cement paste that constitutes the SCC, involves the CRA and penetrates their pore structure.

The chloride migration decreases as the FRA content grows, which was explained by the greater pores filling effect caused by the FRA, since they have a higher content of small particles than river sand [8]. On the other hand, the chloride migration increased with the CRA incorporation ratio [6,7]. This was justified by the greater porosity of the CRA relative to the CNA (because of the hardened mortar part of the RA).

## 2. Experimental Program

### 2.1. Materials

**Figure 1:**
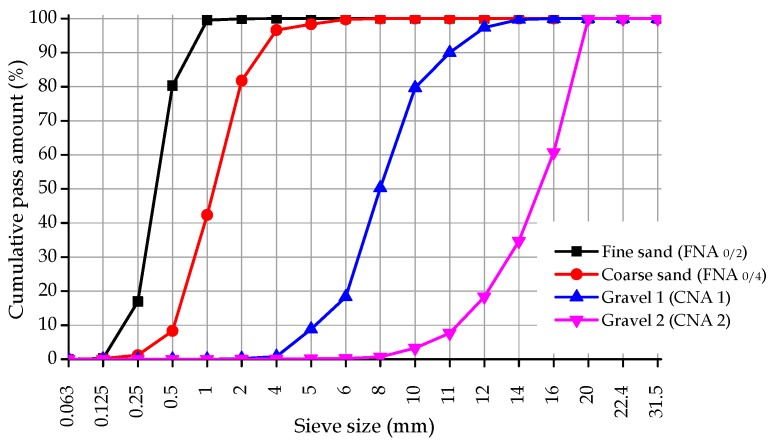
Particle size distribution of the aggregates.

The following materials were used:

- One type of cement complying with NP EN 197-1 [9], cement type CEM I 42.5 R with a density of 3140 kg/m^3^, whose chemical composition is provided in Table 4;
- In order to reduce the amount of cement in the mix, two mineral additions were used: fly ash (FA) complying with NP EN 450-1 [10] and NP EN 450-2 [11] with a density of 2300 kg/m^3^, and limestone filler (LF) complying with specification LNEC-E466 [12] with a density of 2720 kg/m^3^, whose chemical composition is given in Table 4;
- Two limestone coarse aggregates complying with NP EN 12620 [13], gravel 1 with a density of 2640 kg/m^3^, D_max_ of 11 mm and water absorption of 1.60%, and gravel 2 with a density of 2690 kg/m^3^, D_max_ of 20 mm and water absorption of 0.80% (particle size distribution in Figure 1);
- Two siliceous sands complying with NP EN 12620 [13], one coarse (0/4) with a density of 2670 kg/m^3^ and water absorption of 0.40% and one fine (0/2) with a density of 2670 kg/m^3^ and water absorption of 0.40% (particle size distribution in Figure 1);
- The RA came from crushed precast concrete elements of strength classes 45 and 65 MPa, designed to support very long beams: one coarse CRA 65 with a density of 2490 kg/m^3^ and 2.20% water absorption; one coarse CRA 45 with a density of 2600 kg/m^3^ and water absorption of 1.80%, one fine FRA 65 with a density of 2450 kg/m^3^ and water absorption of 7.50% and one fine FRA 45 with a density of 2560 kg/m^3^ and 5.00% water absorption;
- A third-generation high-range/strong water-reducing admixture (Sp) complying with NP EN 934-1 [14] and NP EN 934-2 [15] (a modified polycarboxylic high-range water-reducing admixture in liquid form with a density of 1070 kg/m^3^);
- Tap water complying with NP EN 1008 [16].

The RA were separated according to their size by mechanical sieving, and all fractions were used so the particle size distribution of the NA and RA was the same. Despite this, the grading curves of the NA and RA were different, and therefore, it was necessary to adjust the latter to match the former to achieve a similar fineness modulus. To accomplish this, it was necessary to separate the RA according to their different particle sizes. Although this type of procedure is difficult for practical application, it enables comparisons between mix compositions with the same particle size distribution, even though the replacement ratios differ.

### 2.2. Mix Proportions

To cover all the content alternatives used in the mixes and the corresponding analysis of the RA influence, 10 SCC mixes were produced according to NP EN 206-9 [17]. The mix proportions and basic fresh-state properties of the SCC produced are shown in Table 5.

A preliminary stage of mortar study was carried out, which allowed determining the composition of the various mixes by adjusting the W/C ratio and the superplasticizer content, before adding the coarse aggregate (natural and/or recycled) in order to evaluate, at this stage, the workability parameters.

A value for the ratio, in absolute volume, between the total amounts of fine material (cement and additions) and fine aggregates in the mixes (V_p_/V_s_) was set. According to the results obtained by Silva et al. [18], the value V_p_/V_s_ = 0.80 was considered.

The percentage of cement replacement by additions (f_ad_) was determined taking the target strength into account. For 65 MPa mortars, the value of f_ad_ is necessarily lower than the one assumed for 45 MPa mortars, since mixes with higher strengths require higher percentage of cement in their formulation.

All studied mortar and concrete mixes contemplated the introduction of LF and FA in ternary mixes; for 45 MPa mortars, a 60% value of f_ad_ was considered, corresponding 50% to FA and 10% to LF, and 35% value of f_ad_ for 65 MPa mortars, corresponding 30% to FA and 5% to LF.

The V_w_/V_p_ (ratio between the water and fine material contents, in absolute volume) and S_p_/p% (percentage ratio between the superplasticizer and fine material contents, in weight) values vary according to the need of water and S_p_ of each mix. Since the objective was to maintain the workability constant while maintaining the volume of fine material in each family of self-compacting mortar (45 MPa and 65 MPa) constant, a starting pair of hypothetical values for V_w_/V_p_ and S_p_/p% was considered (V_w_/V_p_ = 0.80 and S_p_/p% = 0.65 for 65 MPa mortars and V_w_/V_p_ = 0.85 and S_p_/p% = 0.50 for 45 MPa mortars) and gradually adapted to the target workability parameters in an iterative process. These parameters were chosen while taking into account several works on SCC using the Nepomuceno et al. [19] method, which presented satisfactory results [2,18,20,21,22], and adjusted afterwards.

The adjustments were only made for the reference mortar (100% NA). Once the proper self-compacting mortar workability was reached, all the method parameters (V_p_/V_s_, f_ad_, V_w_/V_p_ and S_p_/p%) remained constant, and only natural aggregate replacements were made (25%, 50% and 100%). This substitution was made in mass and for each particle size fraction, reproducing the reference curve obtained by Nepomuceno et al. [19]. Adjustments were also made in the mixing water in order to take into account the effect of the water absorption, which is much higher in RA.

Knowing the absolute volume of all components, their densities and, in the case of fine materials, their unit percentages, the mortars’ composition was determined.

For the SCC formulation, it was necessary to set some additional parameters. The value of the void volume (V_v_) was considered constant and equal to 0.03 m^3^, according to the Nepomuceno et al. [19] method. The “mix number” (MN) is defined as the product of V_p_/V_s_ (already arbitrated) and V_m_/V_g_ (ratio between the mortar and coarse aggregates contents in the mix, in absolute volume), and depends on the desired degree of self-compactability. This parameter was set at 1.82, according to the satisfactory results obtained by Silva and de Brito [2]. Knowing the value of MN, the value of V_m_/V_g_ was calculated, which is required to calculate the amounts of coarse aggregates (natural and/or recycled) in the mix and, along with the fixed parameters determined in the mortar phase, it was possible to calculate all mix contents.

### 2.3. Test Methods and Sample Preparation

The test procedure used in the determination of the density of hardened concrete is described in NP EN 12390-7 [23]. This test was performed at 7, 28 and 91 days on the compressive strength cubes.

The test procedure used in the determination of the compressive strength is described in NP EN 12390-3 [24]. This test was performed at 7, 28 and 91 days on 150 mm cubic moulds and at 28 and 91 days on 150 mm diameter × 300 mm high cylindrical moulds.

The test procedure used to determine the splitting tensile strength is described in NP EN 12390-6 [25]. This test was performed on 150 mm diameter × 300 mm high cylindrical moulds at 28 and 91 days.

The test procedure used to determine the secant elastic modulus of elasticity (E_cm_) is described in LNEC E 397 [26]. This test was performed on 150 mm diameter × 300 mm high cylindrical moulds at 28 and 91 days.

The test procedure used to determine the ultrasonic pulse velocity test is described in NP EN 12504-4 [27]. The ultrasonic waves propagation time was evaluated in saturated 150 mm cubic specimens, later used to determine the compressive strength.

As for abrasion resistance, 71 × 71 × 40 mm prisms were tested, using a grinding wheel, according to DIN 52108 [28].

## 3. Results and Discussion

### 3.1. Fresh Concrete Properties

The tests on SCC in the fresh state were performed in order to verify their compliance with the workability parameters required by NP EN 206-9 [17]. The workability of concrete was a parameter fixed a priori through the compositions adjustment previously carried out in self-compacting mortars (Table 5 summarizes the results obtained in the fresh state).

It was noted that, in general, the variations registered in SCC with RA are due to the higher water absorption of the recycled aggregates, affected by the presence of adhered mortar as well as its rougher surface when compared to the NA.

It was concluded that all mixes fulfilled the workability parameters, so they have the characteristics required to be classified as SCC: fluidity; flow speed, either in the absence or the presence of obstructions; filling capacity; flow capacity; ability to pass; and segregation resistance [17,29].

### 3.2. Hardened Concrete Properties

#### 3.2.1. Density

**Figure 2:**
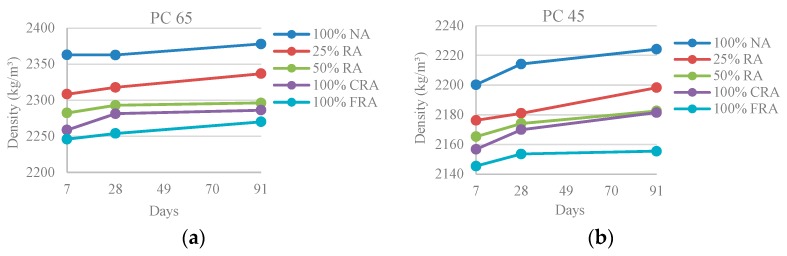
(**a**) Density at 7, 28 and 91 days (PC 65); (**b**) Density at 7, 28 and 91 days (PC 45).

For each mix, it was found that density increases with age (Figure 1 and Figure 2), although not in a very pronounced way (increase of less than 2%, from 7 to 91 days). As the concrete age increases, the more developed its microstructure becomes, filling the existing empty pores and increasing its density [30].

Figure 2 shows that density decreases with the incorporation of RA, due to their lower density relative to NA. The 100%-FRA mixes are those with greater losses of density, with a loss of 5% for PC 65 family and 3% for PC 45 family. The same trend was observed in the study of Pereira-de-Oliveira et al. [4], in which there was a loss of density of 3% in the concrete with 100% RA when compared to the reference SCC.

#### 3.2.2. Compressive Strength

**Figure 3:**
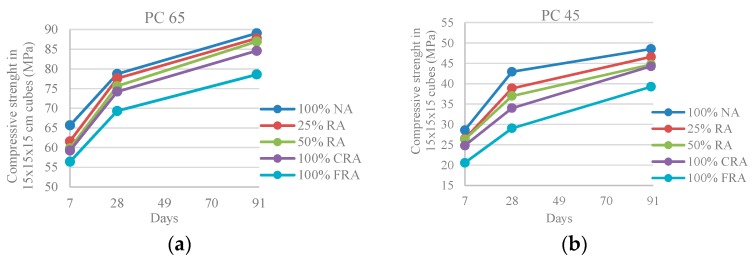
(**a**) Compressive strength in cubes at 7, 28 and 91 days (PC 65); (**b**) Compressive strength in cubes at 7, 28 and 91 days (PC 45).

Figure 3 shows that the replacement of NA with RA causes a reduction of strength, relative to the reference SCC (100% NA), of 2–14% for the PC 65 family and 4–32% for the PC 45 family. The losses are similar at 7, 28 and 91 days. The decrease of this property is due to the flattened and angular shape of the recycled material and the mortar adhered to the RA, which causes an increase of porosity and cracking of the aggregates, weakening the links in the transition zone between the recycled aggregate and new paste [6].

The obtained results show that the reference SCCs (100% NA) present the highest compressive strength, reaching 78 MPa and 43 MPa at 28 days, for the families PC 65 and PC 45, respectively. The SCCs with 100% FRA are those with lower resistance: 69 MPa and 29 MPa, respectively, in the PC 65 and PC 45 family (with 12% and 32% reductions relative to the reference SCC). Therefore, it is concluded that the objective of replicating the strength of the source concrete was only achieved (and exceeded) in the PC 65 family.

**Figure 4:**
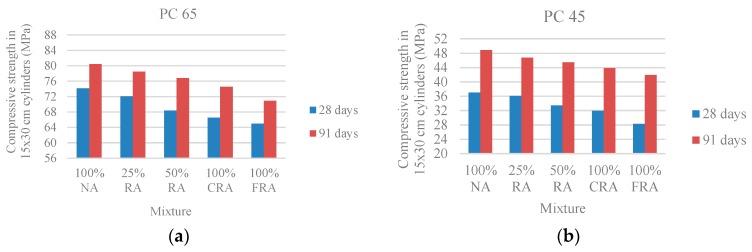
(**a**) Compressive strength in cylinders at 28 and 91 days (PC 65); (**b**) Compressive strength in cylinders at 28 and 91 days (PC 45).

Figure 4 shows a compressive strength reduction with the increasing substitution of NA with RA, as in the compressive strength in cubes, for the same reasons. The variations range from 3% to 12% for the PC 65 family and between 3% and 24% for the PC 45 family. As expected, it was found that the compressive strength increases with age.

**Figure 5:**
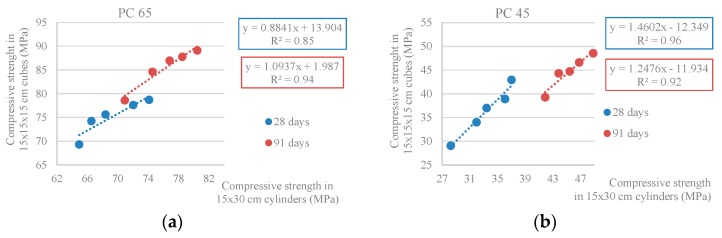
(**a**) Relationship between compressive strength in cubic and cylindrical specimens at 28 and 91 days (PC 65); (**b**) Relationship between compressive strength in cubic and cylindrical specimens at 28 and 91 days (PC 45).

Figure 5 shows the ratio between the compressive strength in cubic and cylindrical specimens, i.e., the conversion factor from 150 mm cubes to cylinders with 150 mm diameter and 300 mm height.

Figure 5 shows the high correlation of the values obtained: R^2^ = 0.85 and R^2^ = 0.94 (PC 65) and R^2^ = 0.96 and R^2^ = 0.92 (PC 45). This conversion factors are near the values presented in Table 7 from NP EN 206-1 [31] that vary from 0.80 (strength class C8/10) to 0.87 (C100/115).

Table 6 and Table 7 show the comparison between the characteristics values of the compressive strength in cubes and cylinders at 28 days and those specified in NP EN 206-1 [31]. The characteristic values of the compressive strength of concrete (f_ck_) were obtained using Equation (1), from Table 3.1 of EC2 [32], where f_cm_ is the average compressive strength of concrete.

f_ck_ = f_cm_ − 8 (MPa) (1)

Comparing the characteristic strength values obtained in cubes and cylinders at 28 days with those specified in NP EN 206-1 [31], it was observed that, for the PC 65 family, the 100% CRA and 100% FRA mixes belong to the C50/60 strength class, while all the others are classified as C55/67. For the PC 45 family, the 100% FRA concrete are classified as C16/20 (the class with the worst performance), the 100% CRA as C20/25 and the others as C25/30. This classification reinforces the former conclusion on the compressive strength test in cubes: the strength of the RAs’ source concrete was replicate only for the PC 65 family. For the PC 45 family, despite not having achieved this goal, all SCCs, except for the 100% FRA mix, belong to the C20/25 strength class or higher, and therefore have application as structural concrete.

#### 3.2.3. Splitting Tensile Strength

**Figure 6:**
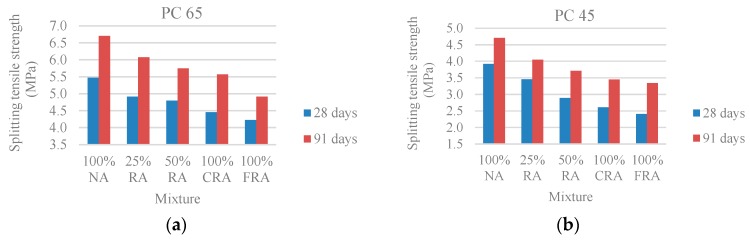
(**a**) Splitting tensile strength at 28 and 91 days (PC 65); (**b**) Splitting tensile strength at 28 and 91 days (PC 45).

Figure 6 shows that the replacement of NA with RA causes a reduction in tensile strength of 9–27% for the PC 65 family and 12–39% for the PC 45 family, when compared to the reference SCC (100% NA), and the concrete with 100% FRA is the one with the lowest strength. This reduction is explained by the mortar adhering to the original natural aggregates [33].

**Figure 7:**
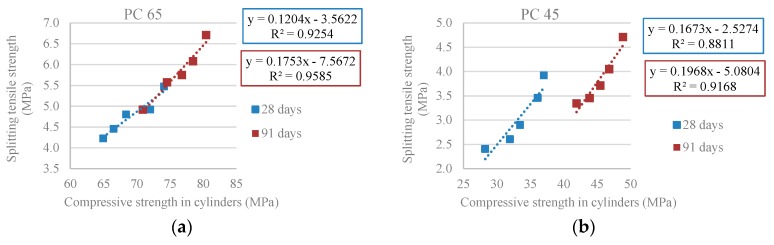
(**a**) Splitting tensile strength versus compressive strength in cylindrical specimens at 28 and 91 days (PC 65); (**b**) Splitting tensile strength versus compressive strength in cylindrical specimens at 28 and 91 days (PC 45).

Figure 7 shows a high correlation between the uniaxial compressive strength and the splitting tensile strength: R^2^ = 0.93 and R^2^ = 0.96 (PC 65) and R^2^ = 0.88 and R^2^ = 0.92 (PC 45), thus allowing concluding that there is a string link between the two properties.

Table 3.1 from EC2 [32] presents a formula (for conventional concrete) to determine the average tensile strength (f_ctm_) based on the average values of the compressive strength obtained experimentally in cylinders (f_cm,cyl_):

f_ctm_ = 2.12 ln(1 + (f_cm,cyl_)/10)) (MPa) (2)

Comparing the results of the tensile strength obtained experimentally with those taken from this equation (Table 8 and Table 9), it is found that they are close. Generally in both families, the experimental splitting tensile strength values are slightly higher than those determined according to EC2 [32].

#### 3.2.4. Ultrasonic Pulse Velocity

**Figure 8:**
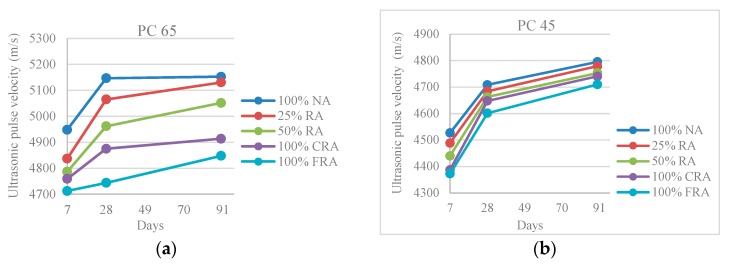
(**a**) Ultrasonic pulse velocity at 7, 28 and 91 days (PC 65); (**b**) Ultrasonic pulse velocity at 7, 28 and 91 days (PC 45).

A decrease in the ultrasonic pulse velocity was observed with the increase of the RA in-corporation ratio (Figure 8), and the reference SCC (100% NA) is the one that presents higher propagation speed, reaching 5152 m/s (PC 65) and 4795 m/s (PC 45), at 91 days. On the other hand, the SCC with 100% FRA has lower pulse velocity, with a loss of 6% (PC 65) and 3% (PC 45) compared to the reference SCC. The reduction of pulse velocity with the incorporation of RA in concrete is explained by the aggregates’ nature, since its porosity in-creases (compared to NA) due to the mortar adhered.

According to the classification suggested by Malhotra [34], all concrete mixes are classified as “good”, since the lower values of ultrasound pulse velocity are included in the range 3660–4580 m/s. Therefore, none of the studied mixes has voids or cracks which may affect its structural integrity.

#### 3.2.5. Secant Modulus of Elasticity

**Figure 9:**
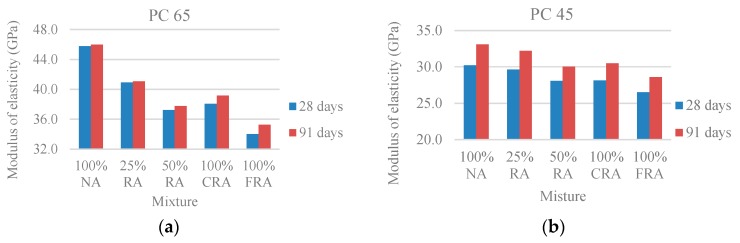
(**a**) Modulus of elasticity at 28 and 91 days (PC 65); (**b**) Modulus of elasticity at 28 and 91 days (PC 45).

Analysing the results (Figure 9), there was a reduction of the modulus of elasticity with the increase of the RA incorporation ratio, registering maximum variations of 26% and 23%, respectively, at 28 and 91 days for the PC 65 family and 12% and 14% for the PC 45 family. Pereira-de-Oliveira et al. [4] obtained a reduction of 5% for 100% RA at 28 days, which is explained by the lower stiffness of the RA (in comparison with the NA) due to the presence of old mortar adhered to the aggregates and with greater deformability than those used in this study [4].

Table 3.1 from EC2 [32] presents a formula (for conventional concrete) to determine the average modulus of elasticity (E_cm_) based on the average values of the compressive strength obtained experimentally in cylinders (f_cm,cyl_):

E_cm_ = 22 ((f_cm,cyl_)/10)^0.3^ (GPa) (3)

Comparing the results of the modulus of elasticity obtained experimentally from the PC 65 family with those taken from this equation (Table 10), it is found that they are close. The mixes with 100% NA and 25% RA have higher modulus of elasticity values than those determined according to EC2 [32], while the remaining mixes have slightly lower values.

**Figure 10:**
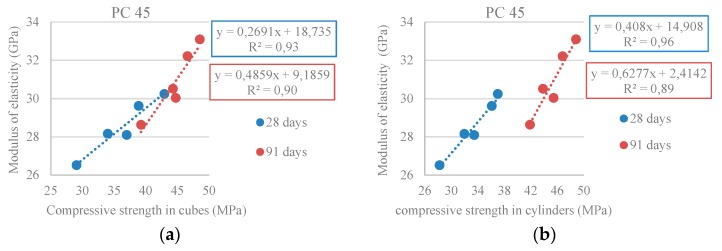
(**a**) Modulus of elasticity vs. compressive strength in cubic specimens at 28 and 91 days (PC 45); (**b**) Modulus of elasticity vs. compressive strength in cylindrical specimens at 28 and 91 days (PC 45).

Figure 10 shows a high correlation between the modulus of elasticity and the compressive strength: R^2^ = 0.93 and R^2^ = 0.90 (at 28 and 91 days) for strength in cubes and R^2^ = 0.96 and R^2^ = 0.89 for strength in cylinders, allowing for the conclusion that there is a strong link between the two properties. In order to limit the volume of data, only the results from the PC 45 family were presented.

#### 3.2.6. Abrasion Resistance

**Figure 11:**
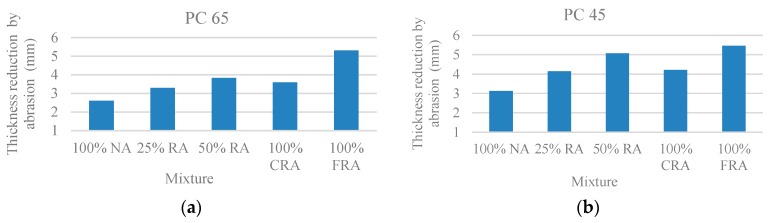
(**a**) Abrasion resistance at 91 days—wear depth (PC 65); (**b**) Abrasion resistance at 91 days—wear depth (PC 45).

Figure 11 shows that there wear depth increases as the ratio of replacement of NA with RA increases, with the exception of the mix with 100% CRA, which presents a lower wear than the mix with 50% RA. The greatest loss of thickness occurs in the SCC with 100% FRA, with a reduction of 105% in the PC 65 family and 75% in the PC 45 family. For conventional concrete, Pereira et al. [35] obtained reductions of 21%, 37% and 50%, respectively for concrete without superplasticizer, with conventional superplasticizer and with high-performance superplasticizer. This loss of resistance is explained by the higher porosity of the RA and consequent lower density, which reduce their stiffness.

## 4. Conclusions

The tests in the fresh state were performed in order to check the compliance of the SCC mixes with the fresh properties required by NP EN 206-9 [17]. As such, the workability of concrete was a parameter fixed a priori through the compositions adjustment previously carried out in self-compacting mortars.

All mixes complied with the requirements demanded from a SCC: fluidity; flow speed, both in the absence and the presence of obstructions; filling capacity; flow capacity; passing ability; and segregation resistance. The concrete density did not significantly change with the incorporation of RA and its variation is closely related with the density of the RA used in each composition.

In terms of the SCC’s mechanical strength, in compression and tension, it is concluded that the replacement of NA with RA causes a performance loss, explained by the lower quality of RA compared to NA, due to the adhered mortar. The strength loss was greater in the PC 65 family, due to the worst quality of the RA, resulting from a lower strength source concrete. The objective of reproducing the strength of the RA’s source concrete was reached only in the PC 65 family. However, for the PC 45 family, all the mixes, except for the one with 100% FRA, belong to the C20/25 strength class or higher, and can thus be used as structural concrete. Besides the RA’s lower strength, the higher W/C ratio may have contributed to the poorer performance of the PC 45 family mixes.

The modulus of elasticity suffered a reduction as the FRA incorporation increased, explained by the latter’s lower stiffness and the greater deformability of the adhered mortar. It was found that the mix with 100% CRA had a better performance than the one with 50% RA, meaning that this property was most influenced by the FRA content. The ultrasonic pulse velocity decreased with the RA incorporation ratio, even though this reduction is small, being caused by the increase in porosity of the RA, again linked with adhered mortar. The abrasion wear increased with the FRA replacement ratio, because of the latter’s greater porosity and consequent lower density, leading to a stiffness loss.

Finally the following general conclusions are presented, based on the results of the experimental work performed:

- The aggregates used, coming from discarded elements from the concrete precasting industry, are of excellent quality, with a better performance in terms of all their properties than that reported in most of the research consulted;
- For the majority of the mechanical properties, incorporation ratios of 25% RA, 50% RA and 100% CRA are viable;
- Therefore, it is considered that an increase of the maximum incorporation ratios of this type of aggregates in existing codes could be contemplated, provided that their quality is demonstrated (as in the case of aggregates from precast concrete elements).

## Figures and Tables

**Table 1:** Coarse Recycled Aggregates (CRA) and Fine Recycled Aggregates (FRA) influence on Self-Compacting Concrete (SCC) fresh-state performance in previous research.

Fresh-State Properties		Replacement		Studies
		CRA	CRA + FRA	
Fluidity and flow speed in the absence of obstructions	Slump-flow diameter	(−) 6.7% (S = 5.0)	-	[3,5,7]
		(+) 3.8%	(+) 4.7% (S = 0.2)	[4,8]
	Slump-flow time	(+) 25.0% (S = 19.1)	-	[3,5,7]
Viscosity, filling ability and ability to pass through small openings	V-funnel flow time	(+) 62.10% (S = 80.43)	-	[4,5,7]
Passing ability through confined spaces	L-box index	(+) 7.6% (S = 2.9)	(+) 8.7% (S = 1.0)	[3,8]
Sieve segregation test	Sieve segregation index	(−) 24.9% (S = 27.1)	-	[3,5]
		-	(+) 24.7%	[8]
Passing ability through confined spaces	J-ring slump flow	(−) 12.9%	-	[5]

**Table 2:** CRA and FRA’s influence on SCC’s 28-day hardened-state performance in previous research.

28-Day Hardened-State Properties	Replacement		Studies
	CRA	CRA + FRA	
Compressive strength	(−) 10.0% (S = 5.3)	(−) 22.3% (S = 6.0)	[3,4,7,8]
Splitting tensile strength	(−) 22.5% (S = 20.5)	(−) 3.10%	[7,8]
Flexural tensile strength	(−) 14.3%	-	[3]
Shrinkage	-	(+) 141.0% (S = 58.0)	[8]

**Table 3:** CRA and FRA’s influence on SCC 28-day durability performance in different investigations.

28-Day Durability Properties	Replacement		Studies
	CRA	CRA + FRA	
Capillary water absorption	(+) 40.9%	-	[7]
	(−) 12%	-	[4]
Water absorption by immersion	(+) 67.3%	-	[3]
Chloride migration	-	(+) 39.0% (S = 14.1)	[8]
	(−) 2.3%	-	[7]

**Table 4:** Chemical composition of the raw materials.

Chemical Composition of Raw Materials (%) *	CEM I	FA	LF
Al_2_O_3_	5.24	24.7	0.13
CaCO_3_	-	-	98.35
CaO	62.71	2.63	-
Cl^−^	0.01	<0.01	-
Fe_2_O_3_	3.17	5.40	0.03
K_2_O	-	1.11	0.02
MgO	2.23	1.01	0.40
Na_2_O	-	0.89	-
SiO_2_	19.59	54.70	0.30
SO_3_	3.13	1.38	-
TiO_2_	-	-	0.01
Insoluble residue	1.37	-	-
Loss of ignition	2.94	5.10	43.80

* The data in this table correspond to indicative values provided by the suppliers. CEM I: cement type I; FA: fly ash; LF: limestone filer.

**Table 5:** Mix proportions and basic fresh-state properties of the SCC produced.

Mix Components		PC 45					PC 65				
		100% NA	25% RA	50% RA	100% CRA	100% FRA	100% NA	25% RA	50% RA	100% CRA	100% FRA
CEM I 42.5 R	(kg/m^3^)	270					437				
Fly ash (FA)	(kg/m^3^)	247					148				
Limestone filler (LF)	(kg/m^3^)	59					29				
Superplasticizer (S_p_)	(kg/m^3^)	3					4				
Water (W)	(L/m^3^)	187					188				
FNA 0/2	(kg/m^3^)	350	262	175	350	-	348	261	174	348	-
FNA 0/4	(kg/m^3^)	348	261	174	348	-	347	260	173	347	-
FRA	(kg/m^3^)	-	167	333	-	667	-	156	312	-	624
CNA 1	(kg/m^3^)	389	292	195	-	389	389	292	195	-	389
CNA 2	(kg/m^3^)	398	299	199	-	398	398	299	199	-	398
CRA	(kg/m^3^)	-	193	385	770	-	-	184	369	737	-
W/C	(−)	0.69					0.43				
W/CM	(−)	0.36					0.32				
W/FM	(−)	0.32					0.31				
**Basic fresh state properties**											
Slump flow (SF)	(mm)	733	690	688	698	685	765	760	700	718	683
T500	(S)	2.5	2.5	2.5	3.0	2.5	2.0	2.5	2.5	3.0	2.5
V-funnel (T)	(s)	7.5	9.0	8.0	7.0	7.0	9.0	9.0	8.0	9.0	11.0
L-box (PL)	(−)	0.80	0.80	0.80	0.83	0.80	0.81	0.84	0.90	0.80	0.80

**Table 6:** Compressive strength classes determined according to NP EN 206-1 [31] (PC 65).

Mix	Experimentally Obtained Values				NP EN 206-1 Values		
	f_cm.cyl_	f_cm.cube_	f_ck.cyl_	f_ck.cube_	Class	f_ck.cyl_	f_ck.cube_
	(MPa)					(MPa)	
100% NA	74.1	78.7	66.1	70.7	C55/67	55	67
25% RA	72.0	77.6	64.0	69.6	C55/67	55	67
50% RA	68.4	75.6	60.4	67.6	C55/67	55	67
100% CRA	66.5	74.2	58.5	66.2	C50/60	50	60
100% FRA	64.9	69.3	56.9	61.3	C50/60	50	60

**Table 7:** Compressive strength classes determined according to NP EN 206-1 [31] (PC 45).

Mix	Experimentally Obtained Values				NP EN 206-1 Values		
	f_cm.cyl_	f_cm.cube_	f_ck.cyl_	f_ck.cube_	Class	f_ck.cyl_	f_ck.cube_
	(MPa)					(MPa)	
100% NA	37.0	42.9	29.0	34.9	C25/30	25	30
25% RA	36.1	38.9	28.1	30.9	C25/30	25	30
50% RA	33.5	37.0	25.5	29.0	C20/25	20	25
100% CRA	32.0	34.0	24.0	26.0	C20/25	20	25
100% FRA	28.3	29.1	20.3	21.1	C16/20	16	20

**Table 8:** Splitting tensile strength versus compressive strength determined according to EC2 [32] at 28 and 91 days (PC 65).

Mix	28 Days			91 Days		
	Average Compressive Strength	Average Splitting Tensile Strength		Average Compressive Strength	Average Splitting Tensile Strength	
		EC2 Value	Experimentally Obtained Value		EC2 Value	Experimentally Obtained Value
	(MPa)			(MPa)		
100% NA	74.1	4.5	5.5	80.5	4.7	6.7
25% RA	72.0	4.5	4.9	78.5	4.6	6.1
50% RA	68.4	4.4	4.8	76.8	4.6	5.7
100% CRA	66.5	4.3	4.5	74.6	4.5	5.6
100% FRA	64.9	4.3	4.2	70.9	4.4	4.9

**Table 9:** Splitting tensile strength versus compressive strength determined according to EC2 [32] at 28 and 91 days (PC 45).

Mix	28 Days			91 Days		
	Average Compressive Strength	Average Splitting Tensile Strength		Average Compressive Strength	Average Splitting Tensile Strength	
		EC2 Value	Experimentally Obtained Value		EC2 Value	Experimentally Obtained Value
	(MPa)			(MPa)		
100% NA	37.0	3.3	3.9	48.9	3.8	4.7
25% RA	36.1	3.2	3.5	46.8	3.7	4.0
50% RA	33.5	3.1	2.9	45.5	3.6	3.7
100% CRA	32.0	3.0	2.6	43.9	3.6	3.5
100% FRA	28.3	2.8	2.4	41.9	3.5	3.3

**Table 10:** Modulus of elasticity versus compressive strength according to EC2 [32] at 28 and 91 days (PC 65).

Mix	28 Days			91 Days		
	Average Compressive Strength	Modulus of Elasticity		Average Compressive Strength	Modulus of Elasticity	
		EC2 Value	Experimentally Obtained Value		EC2 Value	Experimentally Obtained Value
	(MPa)	(GPa)		(MPa)	(GPa)	
100% NA	74.1	40.1	45.8	80.5	41.1	46.0
25% RA	72.0	39.8	40.9	78.5	40.8	41.1
50% RA	68.4	39.2	37.3	76.8	40.6	37.8
100% CRA	66.5	38.8	38.1	74.6	40.2	39.2
100% FRA	64.9	38.6	34.0	70.9	39.6	35.3
